# Estimation of physicochemical properties of 2-ethylhexyl-4-methoxycinnamate (EHMC) degradation products and their toxicological evaluation

**DOI:** 10.1007/s11356-018-1796-6

**Published:** 2018-03-28

**Authors:** Alicja Gackowska, Waldemar Studziński, Edyta Kudlek, Mariusz Dudziak, Jerzy Gaca

**Affiliations:** 1 Faculty of Chemical Technology and Engineering, UTP University of Science and Technology, Seminaryjna 3, 85-326 Bydgoszcz, Poland; 2 Institute of Water and Wastewater Engineering, Silesian University of Technology, Konarskiego 18, 44-100 Gliwice, Poland

**Keywords:** EHMC transformation products, Physicochemical properties, EPI suite, *P*_OV_, LRTP, Toxicity

## Abstract

The organic UV filters, commonly used in personal protection products, are of concern because of their potential risk to aquatic ecosystems and living organisms. One of UV filters is ethylhexyl-4-methoxycinnamate (EHMC) acid. Studies have shown that, in the presence of oxidizing and chlorinating factors, EHMC forms a series of products with different properties than the substrate. In this study, the toxicities of EHMC and its transformation/degradation products formed under the influence of NaOCl/UV and H_2_O_2_/UV systems in the water medium were tested using *Microtox*® bioassay and by observation of mortality of juvenile crustaceans *Daphnia magna* and *Artemia Salina*. We have observed that oxidation and chlorination products of EHMC show significantly higher toxicity than EHMC alone. The toxicity of chemicals is related to their physicochemical characteristic such as lipophilicity and substituent groups. With the increase in lipophilicity of products, expressed as log *K*_OW_, the toxicity (EC_50_) increases. On the basis of physicochemical properties such as vapour pressure (VP), solubility (*S*), octanol-water partition coefficient (*K*_OW_), bioconcentration factor (BCF) and half-lives, the overall persistence (*P*_OV_) and long-range transport potential (LRTP) of all the products and EHMC were calculated. It was shown that the most persistent and traveling on the long distances in environment are methoxyphenol chloroderivatives, then methoxybenzene chloroderivatives, EHMC chloroderivatives, methoxybenzaldehyde chloroderivatives and methoxycinnamate acid chloroderivatives. These compounds are also characterised by high toxicity.

**Electronic supplementary material:**

The online version of this article (10.1007/s11356-018-1796-6) contains supplementary material, which is available to authorized users.

## Introduction

Chemical UV filters are used in personal protection products to protect our skin from harmful UV radiation. They are one of the components of sunscreens, lotions, shampoos, body washes, hair sprays and protective lipsticks (Ji et al. 2013; MacManus-Spencer et al. 2011). They are also added to paints and varnishes because they can prevent polymer degradation or pigmentation (Christiansson et al. 2009; Ferrari et al. 2013). One of the commonly used UV filter is 2-ethylhexyl-4-methoxycinnamate ester (EHMC) (Kikuchi et al. 2011). EHMC shows a high absorbing capacity in the UVB range. The maximum permissible concentration of EHMC in cosmetic products in the European Union cannot exceed 10% (Gilbert et al. 2013). Slightly smaller concentration, i.e. 7.5%, is valid in the USA (Janjua et al. 2008).

The dynamic development of cosmetic industry has resulted in a higher consumption of chemical UV filters. Unfortunately, it has been observed that the chemical UV filters contribute to pollution of environment. EHMC has been detected in surface waters (Straub 2002; Poiger et al. 2004; Tarazona et al. 2010), swimming pool waters (Cuderman and Heath 2007; Santos et al. 2012), drinking water (Loraine and Pettigrove 2006; Diaz-Cruz et al. 2012), wastewater (Damiani et al. 2006; Li et al. 2007; Rodil et al. 2012), sewage sludge (De la Cruz et al. 2012; Zuloaga et al. 2012; Barón et al. 2013) and even in human breast milk and human urine (León et al. 2010). In treated wastewater, EHMC was identified at a level of 120–849 ng/L (Ekpeghere et al. 2016). Continuous and uncontrolled emission of the chemical UV filters into environment, even at low concentrations, is unfavourable as they accumulate in living organisms. EHMC accumulates in aquatic biota of different trophic levels with concentrations of up to 340 ng/g lipids in cormorants (Fent et al. 2010). EHMC is known as endocrine disrupting compound that cause adverse effects on human and wildlife. On this basis, the Commission of the European Union (EU 2015/495) placed EHMC on the list of 17 substances subjected to monitoring (Directive 2008/105/EC of the European Parliament and of the Council). EHMC has estrogenic properties both in vitro and in vivo (Schlumpf et al. 2001).

Recent studies have shown that EHMC under sun and UV irradiation forms transformation products (TPs) (MacManus-Spencer et al. 2011; Rodil et al. 2009; Santiago-Morales et al. 2013; Vione et al. 2015). Under the influence of UV radiation and hydrogen peroxide, EHMC is degraded to compounds which, in the presence of reactive forms of oxygen or chlorine, can produce new products, sometimes even more toxic than the substrates themselves (Sakkas et al. 2003; Gackowska et al. 2014; Gackowska et al. 2016). In turn, in the presence of sodium hypochlorite used to disinfect pool waters, chloroorganic derivatives of EHMC are formed (Nakajima et al. 2009; Santos et al. 2012; Gackowska et al. 2016). Understanding the mechanism of EHMC transformations in the environment and the environmental fate of products of these transformations requires knowledge of their physicochemical properties such as water solubility (*S*), octanol-water partition coefficient (*K*_OW_), vapour pressure (VP) and bioconcentration factors as well as half-life in air, water and soil. Determination of the properties of all products is time-consuming and sometimes difficult to perform. A useful tool for the determination of physicochemical parameters is EPI Suite. It allows estimating the physicochemical properties of all EHMC transformation products identified so far. Based on the calculated parameters and half-lives, the overall persistence (*P*_OV_) and long-range transport potential (LRTP) of all EHMC transformation products were calculated with the Organization for Economic Cooperation and Development (OECD) *P*_OV_ and LRTP Screening Tool (http://www.oecd.org/document/24/0,3746,en_2649_34379_45373336_1_1_1_1,00.htm; Wegmann et al. 2009). The data obtained provide information on potential persistence of the transformation products and possible risks associated with their long-range transport in the environment.

From the toxicological point of view, the toxicity of EHMC degradation products is mostly unknown. There is no data on the environmental risk assessment of EHMC transformation products. In some cases, the toxicity of photolytic mixtures was tested e.g. *Vibrio fischeri* microtox assay for 4-methoxybenzaldehyde that showed higher toxicity than EHMC (Vione et al. 2015). It should be noted that EHMC transformation products are formed at low concentrations in complex matrices. Their separation and isolation is laborious and difficult to perform. Hence, the toxicity assessment of a single product is difficult. The solution is to carry out a biotest for a mixture of compounds. Commonly applied test is Microtox® biotest, which uses natural luminescence of *Vibrio fisheri* exhibiting sensitivity to a wide spectrum of toxic organic and inorganic substances. (Hsieh et al. 2004; Bohdziewicz et al. 2016; Kudlek et al. 2016).

Other tests were carried out using the freshwater crustacean *Daphnia magna* (Rozas et al. 2016) and the saltwater crustacean *Artemia salina* (Vasquez and Fatta-Kassinos 2013).

The aims of the studies were to estimate physicochemical parameters and to model *P*_OV_ and LRTP for EHMC and its transformation products formed in oxidation, chlorination and photodegradation processes, simultaneously, to perform various ecotoxicological bioassays so as to be able to correlate if possible the findings between the physicochemical and biological assessments.

## Experimental

### Materials and methods

#### Materials

Analytical standard of 2-ethylhexyl 4-methoxycinnamate (E-EHMC) (98%) was obtained from ACROS Organics (USA) and was kept in lightproof container at 4 °C. Sodium hypochlorite NaOCl with a nominal free chlorine content of 100 g L^−1^ and H_2_O_2_ (30%) was obtained from POCh (Poland). The toxicity tests: *Microtox®*, *Daphtoxkit F®* and *Artoxkit M®* were purchased from MicroBioTest Inc. (Belgium).

#### Oxidation processes

The experimental oxidation processes were performed in a laboratory glass batch reactor with a capacity of 0.7 L of Heraeus (Hanau, Germany). The reactor was equipped with an immersion medium pressure UV lamp of 150 W located in a cooling jacket made of Duran 50 glass. The cooling process was performed with water from the mains. The cooling process enabled a constant temperature of 20 ± 1 °C to be maintained. The lamp emitted radiation of *λ*_exc_ equal to 313, 365, 405, 436, 546 and 578 nm. Additionally, the reactor was situated on a magnetic stirrer to guarantee the even mixing of contents during the execution process. The reaction conditions are presented in Table 1.

**Table 1:** The reaction conditions and substrate proportions used in this study

Reagents	EHMC [M]	H_2_O_2_ [M]	NaOCl [M]	UV [W]
EHMC	3.4·10^−4^	0	0	–
EHMC/UV	3.4·10^−4^	0	0	150
EHMC/NaOCl/UV	3.4·10^−4^	0	1.7·10^−5^	150
EHMC/H_2_O_2_/UV	3.4·10^−4^	0.05	0	150
NaOCl/UV	0	0	1.7·10^−5^	150
H_2_O_2_/UV	0	0.05	0	150

The research subjects were model solutions containing deionised water and E-EHMC model. In order to test toxicity of the E-EHMC oxidation and chlorination products, E-EHMC solution at concentration of 3.4·10^−4^ M was prepared and subjected to the action of UV only, H_2_O_2_/UV and NaOCl/UV. The concentration of sodium hypochlorite and hydrogen peroxide were respectively 1.7·10–5 M and 0.05 M. After 30, 60, 90 and 180 min, mixtures of the products obtained were sampled from reaction systems and subjected to toxicity tests. The effectiveness of E-EHMC elimination was assessed by monitoring for changes in concentrations of compound in water before and after the oxidation process, respectively.

#### Method for the determination of EHMC transformation products

A GC-MS 5890 HEWLETT PACKARD instrument equipped with column ZB-5MS (0.25 mm × 30 m × 0.25 μm) was used for the identification of the transformation products applying the following chromatographic conditions: injector temperature 250 °C, oven temperature program from 80 to 260 °C at 10 °C/min, from 260 to 300 °C (held for 2 min) at a rate of 5 °C min. Helium was used as a carrier gas. The volume of the sample was 1 μL. Reaction products were identified by comparing recorded MS spectra with standard spectra from NIST/EPA/NIH Mass Spectral Library. The detailed description of the methodology for identification of EHMC transformation products was presented in previous papers (Gackowska et al. 2014; Gackowska et al. 2016).

#### Toxicity tests

All samples from the reactor were diluted 1:100 before performing toxicity tests. Additionally, control tests were carried out. In order to eliminate the effects of the reagents, tests for E-EHMC-free systems were performed. Moreover, the toxicity tests were performed without EHMC. Changes in the toxicity of samples were assessed on the basis of the results from three biotests: *Microtox®*, *Daphtoxkit F®* and *Artoxkit M®*. On the basis of the difference in results obtained for EHMC systems with and without EHMC, the toxicity of the mixture of transformation products was determined. All samples for toxicity tests were performer in four replicates.

##### Microtox®

In *Microtox*® test, bioluminescent bacteria *Aliivibrio fischeri*, which are highly sensitive to a wide spectrum of toxic substances, were used. During exposure of bacteria to toxic substances, the metabolic changes occur or population of bacteria is reduced, what in turn results in change in the intensity of light emitted by microorganisms. The test was conducted according to MicrotoxOmni Screening Test procedure in the *Microtox* Model 500 analyser from Tigret Sp. z o.o. (Poland), which operated both as an incubator and as a photometer. Percentage of bioluminescence inhibition relative to control sample (bacteria not exposed to toxicant) was measured after 5 and 15 min of exposure time (volume of samples 1 mL). The EC_50_ value was determined on the basis of the Basic Dilution Test.

##### Daphtoxkit F®

The test procedure is based on observation of the mortality of juvenile *Daphnia magna* crustaceans subjected to the action of toxicant. The results were checked after 24 and 28 h of exposure of animals to the tested solutions. All organisms that did not demonstrate a motion reaction to swirl induced by stirring the solution were considered dead. Experiment was carried out in accordance with the OECD Guideline 202 and ISO 6341 standards.

##### Artoxkit M®

Toxicity of solutions was also tested on *Artemia Salina* crustaceans. Survival of indicatory organisms was assessed after 24 h of exposure to water solutions. The individuals showing no signs of life were recognised as dead. Test was conducted according to the ASTM E1440-91 standard.

The effect of the toxicity (%) was determined according to the equation:

1 $$ E=\frac{100\bullet \left({E}_{\mathrm{K}}-{E}_{\mathrm{T}}\right)}{E_{\mathrm{K}}},\kern0.5em \left[\%\right] $$

where

*E*_K_: the effect observed in a blank sample and
*E*_T_: the effect observed in a test sample.

Depending on the given test, the effect was measured by the decrease in bioluminescence (i.e. the enzymatic *Microtox®* test) or organism viability (i.e. the *Daphnia magna* test and *Artemia Salina* test).

#### The evaluation of results

The results are the arithmetic average of the four replicates of each experiment. For all the cases, assigned error (estimated based on the standard deviation) did not exceed 5%, so the results are presented in the form of error bars.

## Results and discussion

Based on the analysis of previous studies, the identified products of EHMC transformation have been gathered. These products have been presented in Supplementary (S Figs. 1–8) and the list of products studied was presented in Table 2.

**Table 2:** List of chemicals

No.	Abbreviation	Chemical name
1	E-EHMC	*trans* 2-Ethylhexyl-4-methoxycinnamate
2	EHA	2-Ethylhexyl alcohol
3	4MCA	4-Methoxycinnamic acid
4	4MBA	4-Methoxybenzaldehyde
5	4MP	4-Methoxyphenol
6	1Cl4MB	1-Chloro-4-methoxybenzene
7	1.3DCl2MB	1.3-Dichloro-2-methoxybenzene
8	2-EHCA	2-Ethylhexyl chloroacetate
9	3Cl4MBA	3-Chloro-4-methoxybenzaldehyde
10	Z-EHMC	*cis* 2-Ethylhexyl-4-methoxycinnamate
11	EHMCCl	Chloro-2-Ethylhexyl-4-methoxycinnamate
12	EHMCCl_2_	Dichloro-2-Ethylhexyl-4-methoxycinnamate
13	2.4DClP	2.4-Dichlorophenol
14	2.6DCl1.4BQ	2.6-Dichloro-1.4-benzoquinone
15	1.2.4TCl3MB	1.2.4-Trichloro-3-methoxybenzene
16	2.4.6TClP	2.4.6-Trichlorophenol
17	3.5DCl2HAcP	3.5-Dichloro-2-hydroxyacetophenone
18	3Cl4MCA	3-Chloro-4-methoxycinnamic acid
19	3.5DCl4MCA	3.5-Dichloro-4-methoxycinnamic acid
20	3.5DCl4MBA	3.5-Dichloro-4-methoxybenzaldehyde
21	3Cl4MP	3-Chloro-4-methoxyphenol
22	2.5DCl4MP	2.5-Dichloro-4-methoxyphenol
23	TP_199_	Transformation product
24	TP_307e_	Transformation product
25	TP_307f_	Transformation product
26	TP_305a_	Transformation product
27	TP_305b_	Transformation product
28	TP_305c_	Transformation product
29	TP_305d_	Transformation product
30	TP_305e_	Transformation product
31	TP_305f_	Transformation product
32	TP_469a_	Transformation product
33	TP_469b_	Transformation product
34	DIAMC	2.4-bis-((2Z.4E)-4-Methoxyhepta-2.4.6-trienyl)-cyclobutane-1.3-dicarboxylic acid bis-(3-methyl-butyl) ester
35	TP_581b_	Transformation product

In order to make a preliminary assessment of EHMC transformation products for potential threats to the environment, their characteristic physicochemical parameters were determined using EPI Suite program. The EPI (Estimation Programs Interface) Suite™ is a suite of physical/chemical properties, aquatic toxicity and environmental fate estimation programs jointly developed by the US EPA and Syracuse Research Corp. (SRC). The US EPA develops and uses models based on (quantitative) structure-activity relationships ([Q]SARs) to estimate critical parameters. Structure-activity relationship (SAR) and quantitative structure-activity relationship (QSAR) models are theoretical models that can be used to quantitatively or qualitatively predict the physicochemical, biological (e.g. an (eco) toxicological endpoint) and environmental fate properties of a chemical substance from the knowledge of its chemical structure.

The results were presented in Table 3. Analysis of parameters has shown that EHMC transformation products are characterised by different properties than the substrate.

**Table 3:** Physical–chemical properties of EHMC and its transformation products

No.	Compound	References	Molecular formula	Mol wt [g mol^−1^]	MP [°C]	BP [°C]	S [mg L^−1^]	VP [mmHg]	BCF	Log *K*_OW_ = log *P*	Log *K*_OA_	Log *K*_OC_	Log *K*_AW_	Henry’s LC [mol dm^−3^ atm^−1^]	Half-life air [h]	Half-life water [h]	Half-life soil [h]	*P*_OV_ [days]	LRTP [km]
1	E-EHMC	–	C_18_H_26_O_3_	290.41	99.87	360.54	0.1548	1.38·10^−5^	667.6	5.80	9.938	4.089	− 4.138	29.4	4.17	360	720	43.26	90.80
2	EHA	1, 2	C_8_H_18_O_1_	130.23	− 70	184.6	880	0.185	25.33	2.73	5.69	1.415	− 2.965	44.9	19.4	208	416	23.02	385.20
3	4MCA	1, 3	C_10_H_10_O_3_	178.19	96	317	712	1.6·10^−4^	3.162	2.68	10.19	1.536	− 7.505	19,300	5.02	360	720	41.41	37.37
4	4MBA	1, 2	C_8_H_8_O_2_	136.15	0	248	4290	0.0303	4.521	1.76	6.25	1.367	− 4.489	54,600	10.4	360	720	33.49	204.03
5	4MP	1	C_7_H_8_O_2_	124.14	57	243	40,000	0.0083	3.285	1.58	7.447	2.28	− 5.867	12,200	8.62	360	720	34.36	150.24
6	1Cl4MB	4	C_7_H_7_CIO	142.59	≤ 18	197.5	237	0.409	27.58	2.78	4.796	2.280	− 2.016	4.46	36.1	900	1.8e + 003	40.73	740.0
7	1.3DCl2MB	4	C_7_H_6_Cl_2_O	177.03	< 25	215.67	140	0.164	52.22	3.14	5.825	2.508	− 2.145	3.1	96.4	900	1.8e + 003	67.67	1912.83
8	2-EHCA	4	C_10_H_19_ClO_2_	192.69	− 8.26	207	48.86	0.168	236.2	3.50	3.655	2.632	− 1.736	2.03	24.9	360	720	33.86	514.10
9	3Cl4MBA	1	C_8_H_7_ClO_2_	170.60	42.61	250.91	508.2	0.0176	14.98	2.44	7.058	1.518	− 4.618	130.0	13	900	1.8e + 003	87.91	250.74
10	Z-EHMC	1, 5	C_18_H_26_O_3_	290.41	99.87	360.54	0.1548	1.38·10^−5^	667.6	5.80	9.938	4.089	− 4.138	29.4	4.17	360	720	43.26	90.80
11	EHMCCl	6, 7	C_18_H_25_ClO_3_	324.85	128.01	386.23	0.01943	1.68·10^−6^	661.4	6.45	10.777	4.344	− 4.268	33.0	4.63	900	1.8e + 003	108.13	133.19
12	EHMCCl_2_	4, 6, 7	C_18_H_24_Cl_2_O_3_	359.30	149.44	404.93	0.00437	3.42·10^−7^	1215	7.16	11.559	4.562	− 4.399	25.6	5.65	900	1.8e + 003	108.15	410.66
13	2.4 DClP	4	C_6_H_4_Cl_2_O	163.0	45.0	210.0	4500	0.09	18.04	3.06	7.108	2.856	− 3.756	43.7	242	900	1.8e + 003	99.62	2473.19
14	2.6DCl1.4BQ	4	C_6_H_2_Cl_2_O_2_	176.99	123	268.4	5056	0.00189	1.771	1.23	8.818	1.0	− 7.588	11,500	52	900	1.8e + 003	70.56	93.35
15	1.2.4TCl3MB	4	C_7_H_5_Cl_3_O	211.45	45	227	29.73	0.056	126.7	3.64	5.569	2.726	− 1.929	1.89	121	1.44e + 003	2.88e + 003	113.33	2433.26
16	2.4.6TClP	4	C_6_H_3_Cl_3_O	197.45	69	246	800	0.008	55.12	3.69	7.663	3.074	− 3.973	385	423	1.44e + 003	2.88e + 003	166.36	2977.4
17	3.5DCl2HAcP	4	C_8_H_6_Cl_2_O_2_	205.04	90.66	299.08	258	1.6·10^−4^	3.713	3.26	7.8	2.31	− 4.540	5940	492	900	1.8e + 003	103.71	2663.03
18	3Cl4MCA	1	C_10_H_9_ClO_3_	212.63	109.81	337.48	382.6	3.75·10^−5^	3.162	2.80	10.435	1.75	− 7.635	36,500	6.98	360	720	41.81	37.37
19	3.5DCl4MCA	1	C_10_H_8_Cl_2_O_3_	247.08	128.70	356.76	70.28	8.38·10^−6^	3.162	3.44	11.205	1.973	− 7.765	25,800	8.1	900	1.8e + 003	105.91	93.34
20	3.5DCl4MBA	1	C_8_H_6_Cl_2_O_2_	205.04	63.98	277.85	96.55	0.00271	46.95	3.08	7.829	1.803	− 4.749	132	14.2	900	1.8e + 003	101.28	270.76
21	3Cl4MP	1	C_7_H_7_ClO_2_	158.59	51.00	241.49	3238	0.0103	10.55	2.24	8.238	2.499	− 5.998	151	12.1	900	1.8e + 003	87.81	187.43
22	2.5DCl4MP	1	C_7_H_6_Cl_2_O_2_	193.03	67.83	269.20	623.1	0.00379	13.17	2.88	9.008	2.717	− 6.128	1640	37.2	900	1.8e + 003	100.89	330.32
23	TP_199_	3	C_9_H_10_O_5_	198.18	152.73	371.83	9287	1.35·10^−7^	3.162	0.80	18.901	3.458	− 18.105	2.64·10^8^	1.04	360	720	31.72	37.37
24	TP_307e_	3	C_18_H_26_O_4_	306.41	141.55	395.38	1.221	1.54·10^−7^	2500	5.32	13.441	4.308	− 8.121	19,700	1.06	360	720	43.27	846.70
25	TP_307f_	3	C_18_H_26_O_4_	306.41	141.55	395.38	0.5314	1.54·10^−7^	1588	5.07	13.191	4.308	− 8.121	8560	3.75	360	720	43.26	634.76
26	TP_305a_	3	C_18_H_26_O_4_	304.39	124.33	383.31	7.226	2.17·10^−6^	154.6	3.75	11.306	3.031	− 7.556	8310	4.09	900	1.8e + 003	107.01	93.33
27	TP_305b_	3	C_18_H_26_O_4_	304.39	129.46	389.96	2.402	1.31·10^−6^	417.5	4.31	11.609	3.155	− 7.299	4580	2.86	900	1.8e + 003	100.75	93.35
28	TP_305c_	3	C_18_H_26_O_4_	304.39	90.85	348.94	2.186	3.24·10^−5^	454.6	4.36	9.312	3.217	− 4.952	168	4.51	900	1.8e + 003	107.71	92.91
29	TP_305d_	3	C_18_H_26_O_4_	304.39	129.46	389.96	2.402	1.31·10^−6^	417.5	4.31	11.609	3.155	− 7.299	4580	3.02	900	1.8e + 003	107.82	101.10
30	TP_305e_	3	C_18_H_26_O_4_	304.39	124.33	383.11	7.226	2.17·10^−6^	154.6	3.75	11.306	3.05	− 7.556	8310	3.82	900	1.8e + 003	107.01	93.34
31	TP_305f_	3	C_18_H_26_O_4_	304.39	124.33	383.31	7.226	2.17·10^−6^	154.6	3.75	11.306	3.06	− 8.121	8310	3.75	360	720	43.09	44.32
32	TP_469a_	3	C_28_H_36_O_6_	468.60	246.19	571.92	0.012	1.58·10^−12^	56.23	6.27	19.064	3.817	− 12.794	4.23·10^10^	2.93	900	1.8e + 003	108.14	2373.53
33	TP_469b_	3	C_28_H_36_O_6_	468.60	246.19	571.92	0.012	1.58·10^−12^	56.23	6.27	19.064	3.817	− 12.794	4.23·10^10^	2.93	900	1.8e + 003	108.14	2373.53
34	DIAMC	8	C_30_H_40_O_6_	496.65	243.43	566.01	0.009	2.42·10^−12^	5410	5.76	17.679	3.644	− 11.919	5.76·10^5^	2.68	900	1.8e + 003	108.123	1718.79
35	TP_581b_	2, 3, 8	C_36_H_52_O_6_	580.81	269.42	621.64	1.057·10^−5^	4.12·10^−14^	15.03	8.56	19.742	5.167	− 11.182	3.36·10^5^	2.18	1.44e + 003	2.88e + 003	173.03	2857.92

1 Gackowska et al. (2014), 2 MacManus-Spencer et al. (2011), 3 Jentzsch et al. (2016), 4 Gackowska et al. (2016), 5 Serpone et al. (2002), 6 Nakajima et al. (2009), 7 Santos et al. (2013), 8 Rodil et al. (2009)

### Boiling point and vapour pressure

Boiling point (BP) and vapour pressure (VP) are the parameters that provide information on whether the compounds, after entering the environment, will evaporate into the atmosphere relatively quickly. Studies have shown that EHMC transformation products can be classified as medium- or low-volatility compounds (BP > 184 °C). Medium-volatility compounds are: EHA; 1Cl4MB; 1,3DC2MB and 2EHCA (BP 184–216 °C). The above-mentioned products are also characterised by the highest vapour pressure value, which ranges from 0.164 to 0.409 mmHg. Other products TP_469a_, TP_469b_, DIAMC and TP_581b_ belong to the group of low-volatility compounds. On the basis of the BP and VP, these transformation products have no predisposition to evaporate and be in gas phase (Table 3).

### Water solubility

High solubility in water suggests that pollutants can migrate with water over long distances. Hydrophilic compounds also have the ability to be readily absorbed by plants. These pollutants can be phytotoxic by damaging shoots and roots, reducing plant growth and disturbing transpiration (Yu-Hong and Yong-Guan, 2006). In turn, pollutants with low solubility can accumulate in sediments.

**Fig. 1:**
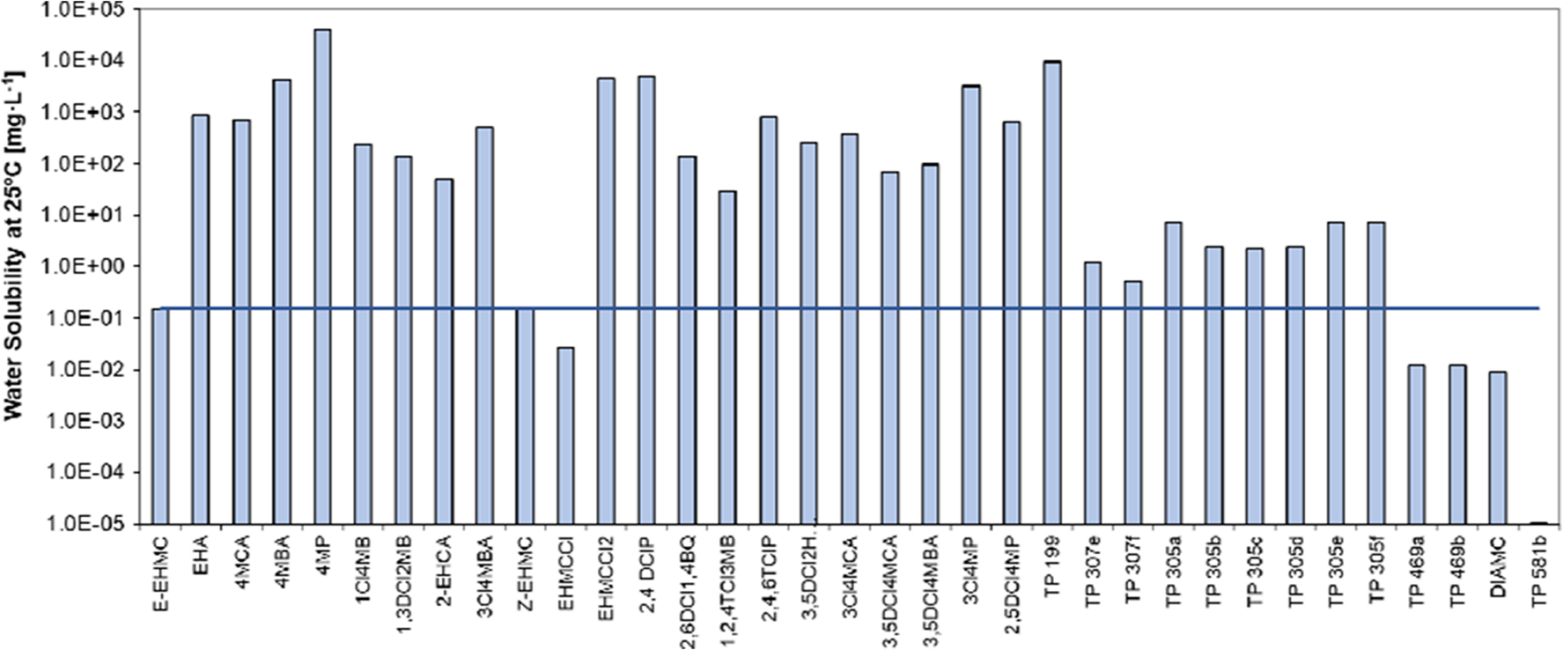
Water solubility of EHMC transformation products

Analysis of the results indicates that the products (besides Z-EHMC, EHMCCl, TP_469a_, TP_469b_, DIAMC and TP_581b_) are characterised by significantly better water solubility than the substrate (Fig. 1). Water solubility of EHMC at temperature of 25 °C is lower than 0.1548 mg L^−1^. Considerably higher solubility (1.0 × 10^3^ ≥ *S* ≤ 1.0 × 10^2^) has the following oxidation products: EHA and 4MCA, and chlorination products: 1Cl4MB; 1,3DCl2MB; 3Cl4MBA; 2,4,6TCP; 3,5DCl2HAcP; 3Cl4MCA and 2,5DCl4MP. Metabolites very well soluble in water (*S* ≤ 1.0 × 10^4^ mg L^−1^) are 4MBA; 4MP; 2,4DClP; 2,6DC1,4BQ; 3Cl4MP and TP_199_. It should be noted that compounds with an OH and Cl group have high *S* values. This pattern indicates that the partitioning potential from water to air of such chemicals is quite low. Among EHMC transformation products, 2,4-dichlorophenol (2,4DClP), 2,4,6-trichlorophenol (2,4,6TClP) and benzene chloroderivatives deserve special attention. Due to their high toxicity to aquatic organisms (USEPA 1991; EC 2001; Xing et al. 2012;) and potentially carcinogenic properties, the international environmental organisations (WHO, UNEP, USEPA, EC) included chlorophenols into a group of pollutants with a special risk to the environment (WHO 1989; WHO 2003; UNEP 2001; USEPA 1991, USEPA 2014; EC 2001). These compounds were identified in surface water and groundwater (He et al. 2000; Czaplicka 2004; Gao et al. 2008; Sim et al. 2009). An example of drinking water pollution with chlorophenol (including 2,4,6TClP) in Finland shows how many effects can be caused by EHMC transformation products, where an increased incidence of gastrointestinal infections, asthma and depression morbidity was observed (Lampi 1992).

### Octanol/water partition coefficient

**Fig. 2:**
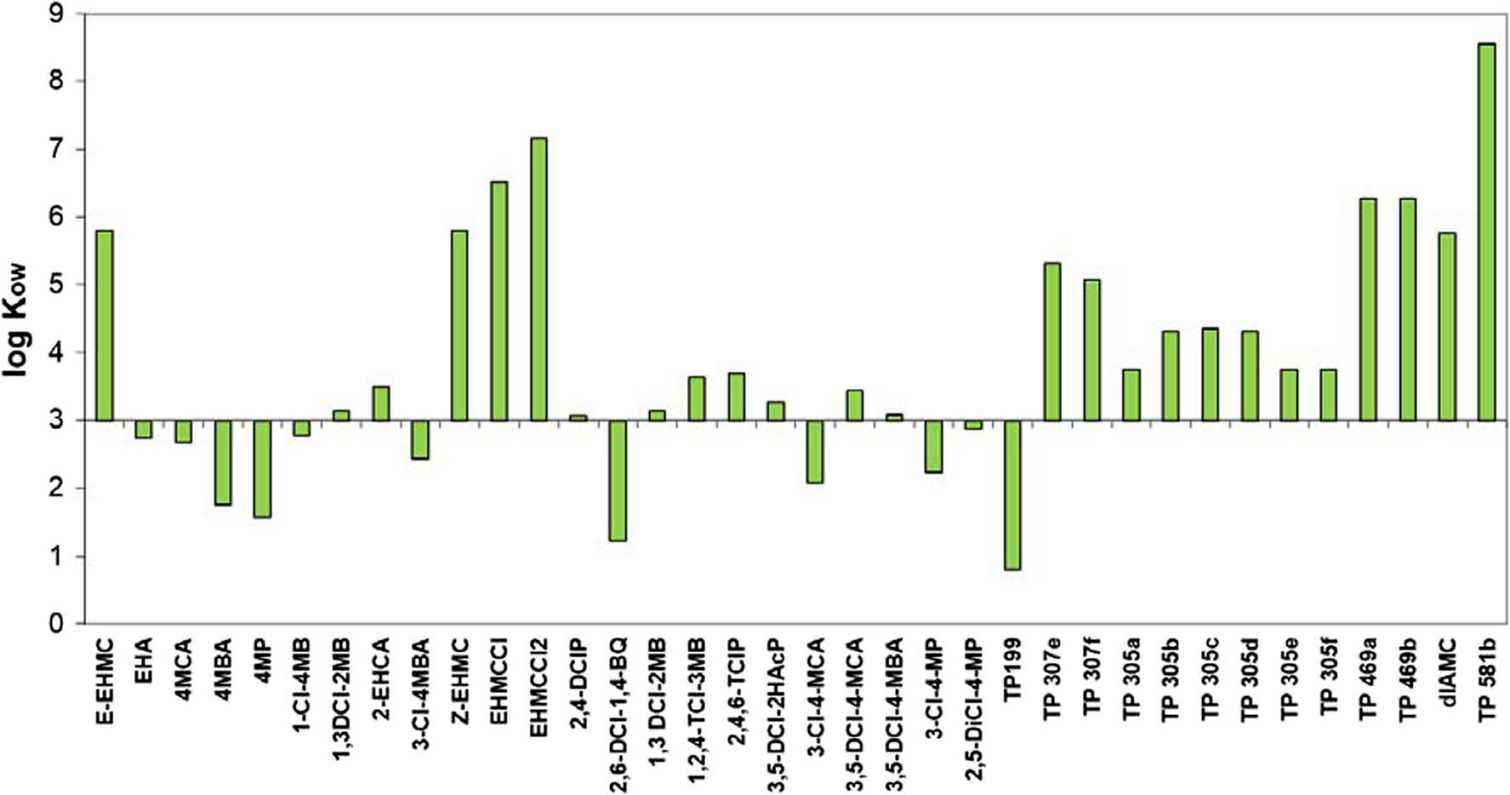
Octanol/water coefficient (log *K*_OW_) of EHMC transformation products

Logarithmic value of octanol/water partition coefficient (log *K*_OW_) allows determining quantitatively lipophilic character of the compound. Octanol is considered as a representative of organic matter. Analysis of the results obtained showed that log *K*_OW_ EHMC was higher than 5 (Fig. 2). The value obtained is consistent with the data presented by Ramos et al. (2015). EHMC has lipophilic properties and can accumulate in sediments. Kupper et al. (2006) and Liu et al. (2012) showed that EHMC concentration in raw sludge is within the range from 13 to 14.45 ng/g dw; however, Langford et al. (2015) reported that it was up to 4689 ng/g dw in treated sludge. The differences in concentration among authors is due to the variable composition of the sludge used, and more likely results from the variable organic matter content they had.

A similar lipophilic character has most of the analysed products for which the calculated coefficient log *K*_OW_ > 3. EHMCCl, EHMCCl2, TP_469a_, TP_469b_ and TP_581b_ for which log *K*_OW_ > 6 deserve a special attention. A different character have the products of EHMC oxidation: EHA; 4 MCA; 4MP; 3Cl4MBA; 2,6DCl1,4BQ; 1Cl4MB; 3Cl4MCA; 3Cl4MP; 2,5DCl4MP and TP_199_ (Fig. 2). Soluble compounds (log *K*_OW_ < 3) will not accumulate in organisms, soil or sediments but instead will be contaminating all water sources and thus spreading around larger areas. Cinnamic acid derivatives with high log *K*_OW_ values show high phytotoxic potential (Jitareanu et al. 2011). According to Legierse et al. (1998), the rate of absorption of chloroderivatives by snails is directly proportional to log *K*_OW_.

### Bioconcentration factor

**Fig. 3:**
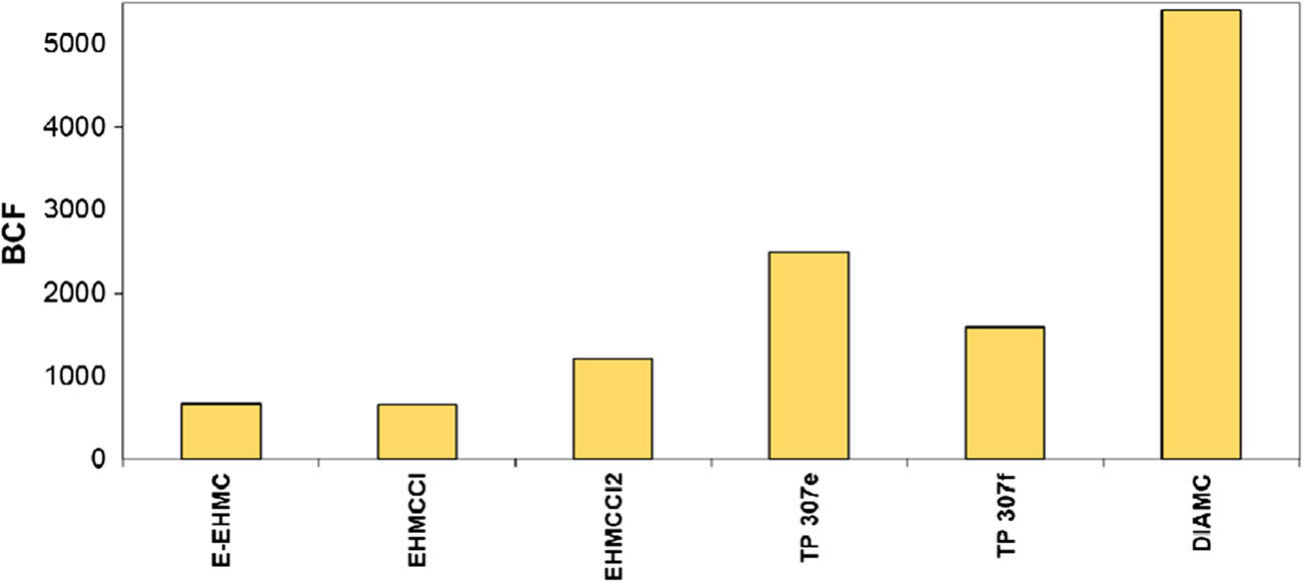
Bioconcentration factor (BCF) of EHMC transformation products with the highest BCF value

The ability of pollutants to bioconcentrate in living organisms is one of parameters taken into account in assessing a threat posed by the new environmental pollutants. For many compounds, there is a linear relationship between log *K*_OW_ and bioconcentration factor (BCF), but this is not a rule, and each example should be considered separately (Axelman et al. 1995). Analysis of products showed that EHMC chloroderivatives (EHMCCl and EHMCCl_2_) were characterised by high bioconcentration factor (BCF > 600) (Fig. 3). These are compounds with hydrophobic properties (log *K*_OW_ > 5). It is accepted that adipose tissue of living organisms is the place where the hydrophobic organic compounds are accumulated. Hydrophobicity is the principal determining factor of bioconcentration and plays a very important role in the bioconcentration of hydrophobic organic compounds (Wang et al. 2014). Hydrophilic compounds appear instead in soluble phases inside the organisms, such as blood serum and mother’s milk (Armitage et al. 2013). They appear also in eggs (Lopez-Antia et al. 2017). They affect not only animals but also plants, where they appear in all plant tissues, including sap and nectar, and thus constitute a major problem in environmental contamination nowadays (Bonmatin et al. 2015). BCF of analysed products with hydrophylic character is in the range of 1.7 < BCF < 56. These include chloroderivatives of phenols, methoxybenzene or methoxycinnamic acid. For this group of compounds, no distinct relationship between log *K*_OW_ and BCF was observed.

The bioconcentration ability of EHMC was confirmed by Fent et al. (2010). EHMC was identified in fish, cormorants and shellfish on a level of nanograms per gram and chlorophenols were present in urine, umbilical cord blood and mother’s milk (Sandau et al. 2002; Bradman et al. 2003; Hong et al. 2005; Philippat et al. 2013; Kim et al. 2014; Forde et al. 2015). These compounds can cause unfavourable histopathological, genotoxic, mutagenic and carcinogenic effects in humans and animals (Igbinosa et al. 2013). Other metabolites that accumulate in the food chains and are ultimately identified in human adipose tissue, breast milk and blood are chlorobenzenes (Ivanciuc et al. 2005; Tor 2006; Kozani et al. 2007). Because EHMC transformations result in formation of many chloroorganic compounds at low concentrations, it should be checked how BCF of the mixture of products will change. According to Kondo et al. (2005), BCF of the mixture of chloroorganic compounds can be significantly higher than that of a single substance.

### Overall persistence and long-range transport potential

As the environmental overall persistence (*P*_OV_) and long-range transport potential (LRTP) of all transformation products cannot be determined in laboratory experiment, they have to be calculated utilising physical–chemical parameters such as n-octanol/water (log *K*_OW_), n-octanol/air (log *K*_OA_) and air/water (log *K*_AW_) partition coefficients, as well as half-lives in air, water, and soil and molar masses of compounds calculated by EPI Suite (Mackay and Webster 2006; Mostrąg et al. 2010; Kuramochi et al. 2014). *P*_OV_ and LRTP of all the products and EHMC were calculated by *P*_OV_ and LRTP Screening Tool created by OECD. The tool requires estimated degradation half-lives in soil, water and air, and partition coefficients between air and water and between octanol and water as chemical specific input parameters. From these inputs, the tool calculates metrics of *P*_OV_ and LRTP from a multimedia chemical fate model and provides a graphical presentation of the results.

**Fig. 4:**
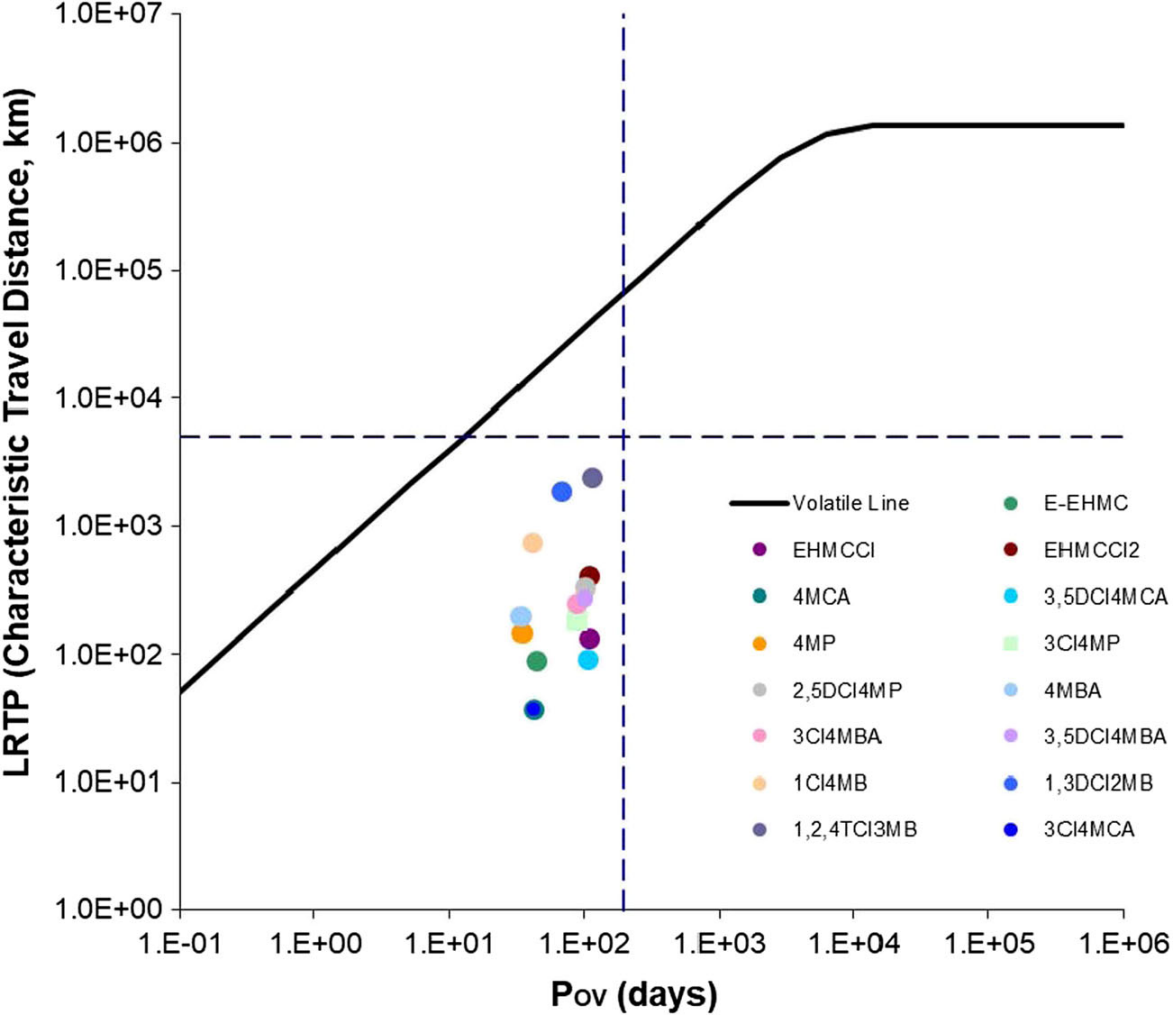
*P*_OV_ and LRTP of the selected EHMC transformation products calculated by the OECD *P*_OV_ and LRTP Screening Tool using property date from EPI Suite

Studies on the environmental mobility of products showed that the highest long-range transport potential expressed by characteristic travel distance (CTD) was observed for methoxyphenol chloroderivatives, then methoxybenzene chloroderivatives, EHMC chloroderivatives, methoxybenzaldehyde chloroderivatives and methoxycinnamate acid chloroderivatives (S Fig. 9). It was observed that CTD increases with the increase of chlorine atoms in molecule. The impact of the compound structure, molar mass and type of atom in the individual molecules was described by Mostrąg et al. (2010). In their opinion, there is a relationship between the long-range transport potential of pollutants and presence of halogens (Cl, F, Br) in the molecule. However, each group of compounds should be analysed individually. Other products that can be transported over considerable distances in the environment are photodegradation products formed by the path of dimerization (TP_469a_, TP_469b_, TP_581b_, dIAMC) (Vione et al. 2015). These compounds can travel up to 3000 km in the environment (Table 3). EHMC oxidation products (4MBA, 4MP, TP_305a–f_) can be transported over much shorter distances. Similar relationships are observed in the case of the overall persistence. The most durable are chloroorganic products. *P*_OV_ of these compounds is in the range of 100–170 days. Similarly, EHMC oxidation products (TP_305a–f_) are also stable (S Fig. 10). On the basis of LRTP and *P*_OV_ values obtained, it can be determined to which class of persistent organic pollutants (POPs) the tested products are classified. Klasmeier et al. (2006) determined the critical values of LRTP and *P*_OV_ and divided pollutants into four classes: I class—persistent organic pollutants (POP-like) (pollutants of the “highest priority”), both parameters are higher than the critical value; II and III classes—molecules which have POP-like characteristic for one of the reference parameters, (pollutants of “intermediate priority”) and IV class—pollutants with LRTP and *P*_OV_ lower than critical value (compounds of the “lowest priority”). LRTP and *P*_OV_ values of the products studied are lower than the critical value (*P*_OV_—195 days, LRTP—5096.73 km); therefore, they can be classified into IV class (Fig. 4).

### Toxicity testing

Toxicity of products was estimated by monitoring changes in the natural emission of the luminescent bacteria *Aliivibrio fisheri* and by observation of mortality of juvenile crustaceans *Daphnia magna* and *Artemia Salina* treated with solutions containing EHMC transformation products. The reaction mixtures EHMC/UV, EHMC/H_2_O_2_/UV and EHMC/NaOCl/UV were tested after different times of reaction (S Figs. 11–17). In order to eliminate the effects of reagents, tests for reaction systems with/without EHMC were performed. Based on the difference in results obtained, the toxicity of the mixture of transformation products was determined.

**Fig. 5:**
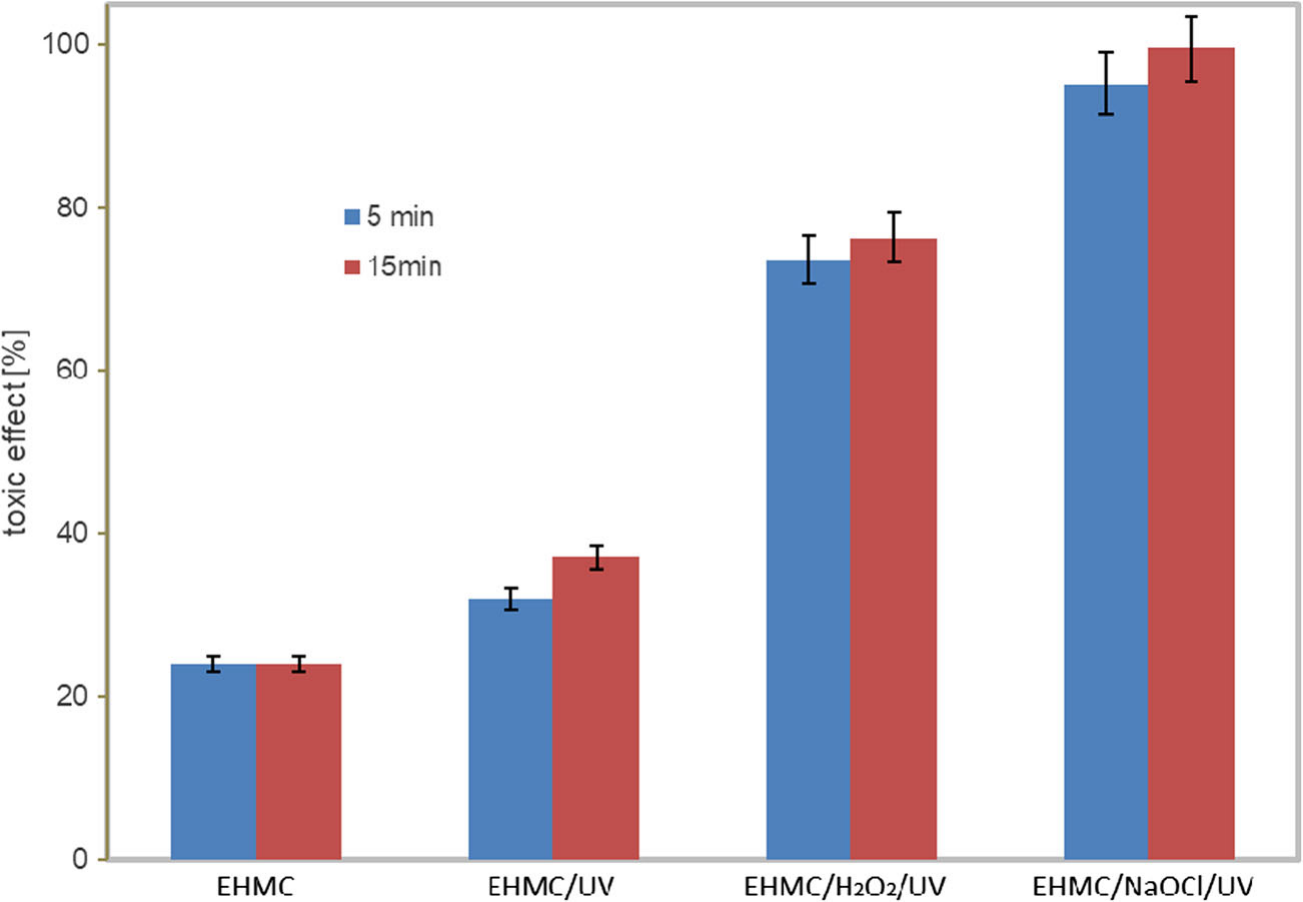
Toxic effect of the systems studied, determined by *Microtox*® test after 90 min of reaction

Analysis of solutions from systems containing only oxidizing agents (NaOCl/UV, H_2_O_2_/UV) showed a slight toxic effect (S Figs. 11 and 12). After an hour of reaction, the toxic effect is close to zero. Figure 5 presents percentage of toxic effect of the systems studied (EHMC, EHMC/UV, EHMC/H_2_O_2_/UV, EHMC/NaOCl/UV), determined by *Microtox*® test after 90 min of reaction. The toxicity classification of the mixture of products was performed based on the magnitude of effects observed in the indicator organisms. The toxicity classification system is presented in Table 4. Such a system is used by many researchers (Põllumaa et al. 2004; Ricco et al. 2004; Werle and Dudziak 2013). EHMC is characterised by low toxicity; toxic effect is lower than 30% (S Figs. 13 and 14). The acute toxicity shows the products formed as a result of EHMC reaction with NaOCl and UV. After 1.5-h-lasting reaction, toxic effect is higher than 90%. In the system with hydrogen peroxide and UV, the toxic products are formed. The effect is on the level of 75%. Low toxicity was observed in the system in which EHMC was exposed to UV. Toxic effect was about 30%. Similar results were obtained using tests with *Daphnia manga* and *Artemia Salina* (S Figs. 15–17). Studies have shown that the presence of oxidizing and chlorinating agents affects the increase of toxicity of EHMC photodegradation products. A similar effect of additional factors was observed by Vione et al. (2015). They have found that in the presence of TiO_2_ and UV, toxicity of photoproducts increased by 40–50% with respect to EHMC.

**Table 4:** Sample toxicity classification system (Ricco et al. 2004; Põllumaa et al. 2014)

Toxicity [%]	Classification
< 25	Not toxic
25–50	Low toxicity
50.1–75	Toxicity
75.1–100	High toxicity

A distinct increase in toxicological response of products, in the case of hydrogen peroxide and sodium hypochlorite, can be explained by formation of cinnamic acid derivatives, among others (esters, aldehydes and alcohols). These compounds have strong toxic action for some bacterial and fungal species (Narasimhan et al. 2004; Guzman 2014). The highest toxicity in the EHMC/NaOCl/UV system can be attributed to formation of chloroorganic products. On the example of chlorophenols and chlorobenzene, it was found that the toxicity increases with the increase in the number of chlorine atoms in molecule (Pepelko et al. 2005; Zhang et al. 2016). The difference in results between the Microtox® (bacteria) and the other two kits is due to the higher sensitivity of water crustaceans (both Daphnia and Artemia) (S Figs. 13–17).

**Fig. 6:**
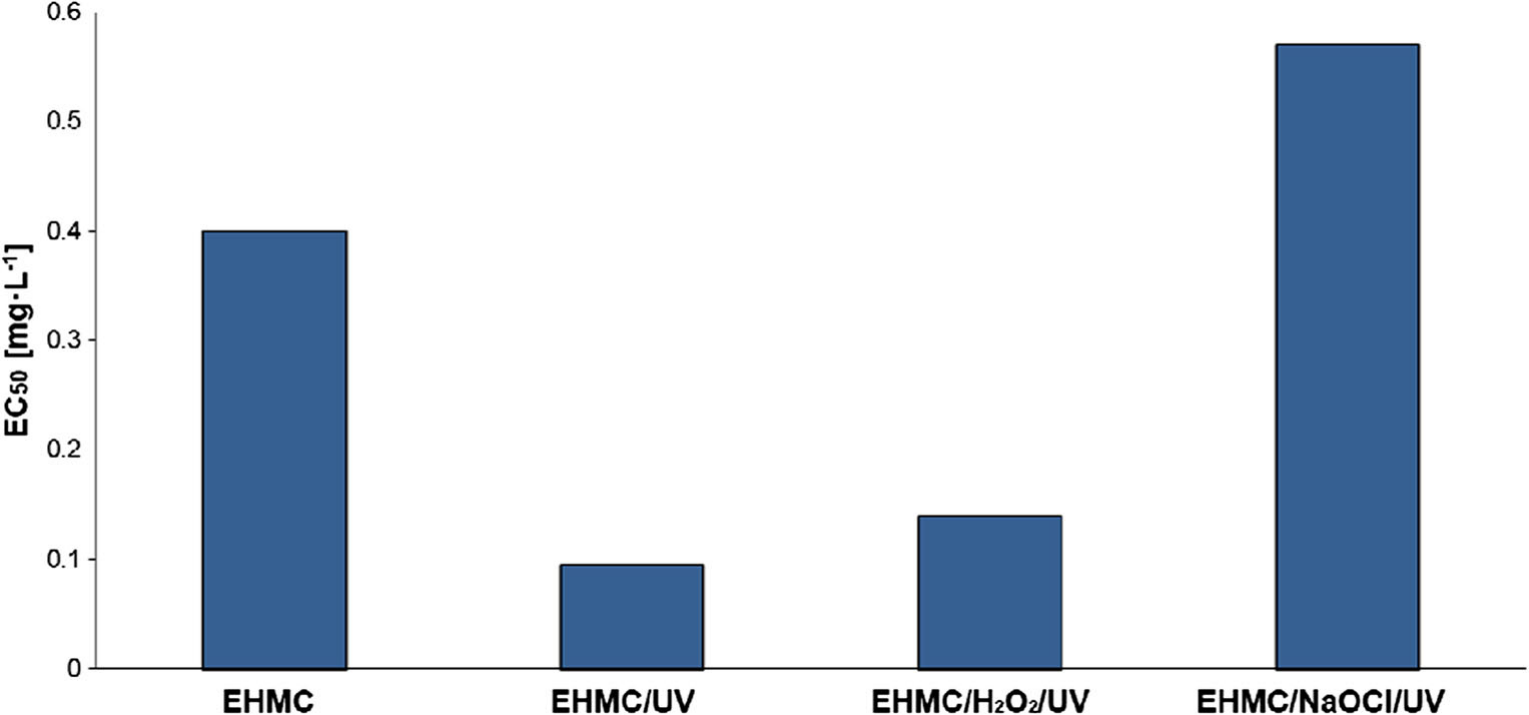
EC_50_ concentration of the systems studied (determined by *Microtox*® after 180 min of reaction)

Moreover, toxicological potential of the tested systems expressed by EC_50_, calculated in milligrams per liter, was evaluated (Fig. 6). EC_50_ value was 0.15 mg L^−1^ for EHMC/H_2_O_2_/UV and 0.094 mg L^−1^ for EHMC/NaOCl/UV, respectively. These values are significantly lower than EC_50_ obtained for EHMC (0.4 mg L^−1^) using bacteria *Aliivibrio fisheri.*

## Conclusions

As a result of the EHMC transformations, a number of products with different properties other than the substrate are produced. Two main classes of EHMC degradation products have been identified. The first includes oxidation products, which due to their hydrophilic character disperse in water, and some of them can evaporate into the air. Whereas, the second class includes chloroorganic products that probably disperse in air and water and can accumulate in an adipose tissue of living organisms. Both of them can reach anywhere on the planet, so both are a cause of concern. However, it is only their persistence and toxicity that can make them problematic. Oxidation products are characterised by a relatively low durability and small range of dispersal in the environment. Much more harmful to the environment are EHMC chlorination products. Based on the guidelines established in Convention Stockholm (2001), the identified chloroorganic products show the properties of persistent organic pollutants. Degradation half-lives of more than 60 days in water or 180 days in soil, respectively, are used to identify chemicals with high potential to be persistent in the environment, and a half-life of longer than 2 days in air is the screening criterion for atmospheric LRTP (Klasmeier et al. 2006). Products such as chlorobenzene and chlorophenol derivatives have tair1/2 values longer than 2 days and tsoil1/2 values longer than 6 months. In addition, they are the compounds with proven mutagenic and carcinogenic effect in humans and animals (Igbinosa et al. 2013). Comprehensive risk assessment also included studies on toxicity of the products formed. We observed that oxidation and chlorination products of EHMC show significantly higher toxicity than EHMC alone. It was found that chloroorganic products are a greater environmental hazard. They are characterised by higher toxicity in the environment than oxidation products.

The results obtained can be a valuable information in the context of assessing the quality of water resources, especially in countries where water shortages are replenished by treated sewage. Incomplete removal of EHMC in conventional wastewater treatment plants (Ekpeghere et al. 2016) indicates that this compound is recalcitrant and contaminates the environment. Analysis of the risk of environmental pollution by new pollutants and their transformation products can be useful in assessing water quality in order to ensure maximum safety for water resources.

## Electronic supplementary material

ESM 1 (DOC 1126 kb)
