# Supplementary material for: Estimation of physicochemical properties of 2-ethylhexyl-4-methoxycinnamate (EHMC) degradation products and their toxicological evaluation
Source: Environ Sci Pollut Res Int. 2018 Mar 28;25(16):16037–49. doi: 10.1007/s11356-018-1796-6 (PMC5984635; doi:10.1007/s11356-018-1796-6)
Supplement: Supplementary file 1 — (DOC 1126 kb) [file 11356_2018_1796_MOESM1_ESM.doc]

**Estimation of physicochemical properties of 2-ethyl-hexyl-4-methoxycinnamate (EHMC) degradation products and their toxicological evaluation**

Alicja Gackowska^a^, Waldemar Studziński^a^, Edyta Kudlek^b^ Mariusz Dudziak^b^ and Jerzy Gaca^a^

^a^Faculty of Chemical Technology and Engineering, UTP University of Science and Technology, Seminaryjna 3, 85-326 Bydgoszcz, Poland;

^b^Institute of Water and Wastewater Engineering, Silesian University of Technology, Konarskiego 18, 44-100 Gliwice, Poland

Fig. 1-4 EHMC transformation products


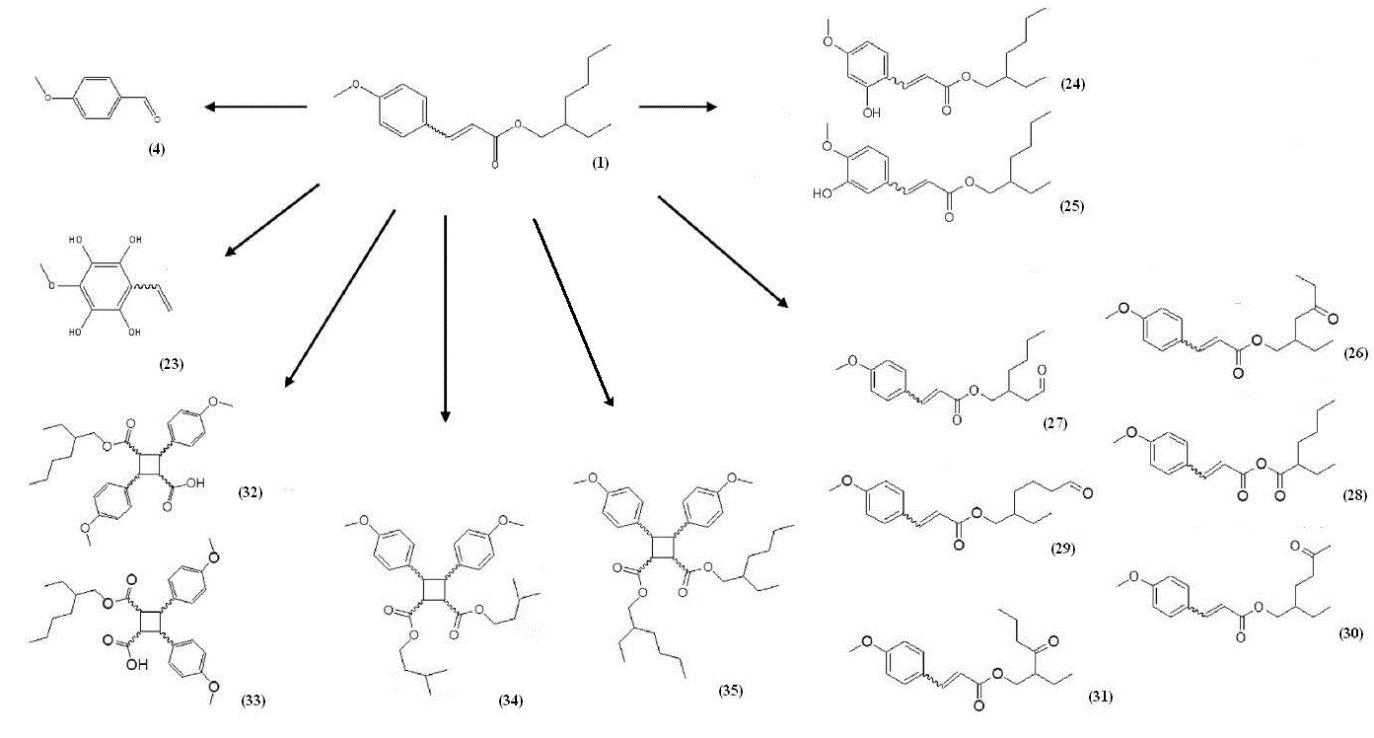


Fig. 1 EHMC photodegradation products in water environment (Serpone et al. 2002; Rodil et al. 2009, MacManus-Spencer et al. 2011; Jentzsch et al. 2016)


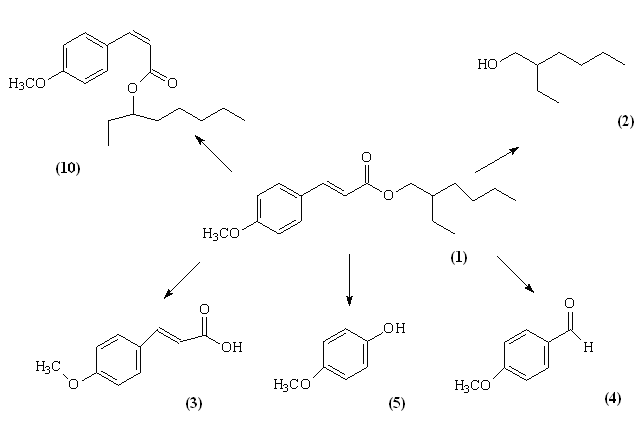


Fig. 2 EHMC oxidation products in the presence of H_2_O_2_/UV (Gackowska et al. 2014)


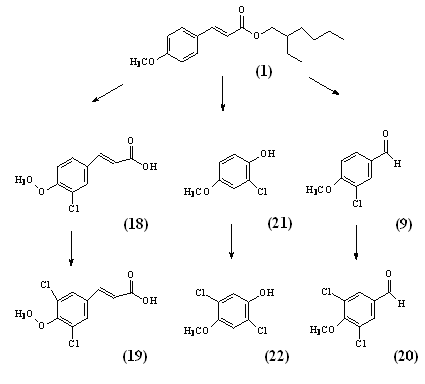


Fig. 3 EHMC oxychlorination products in the presence of HCl/H_2_O_2_/UV (Gackowska et al. 2014)


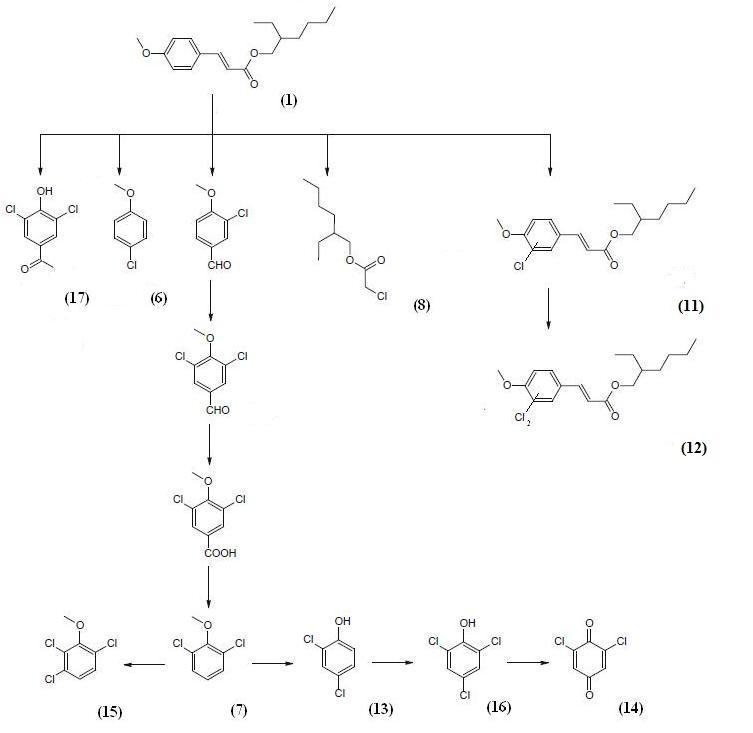


Fig. 4 EHMC chlorination products in the presence of NaOCl/UV (Negreira et al. 2008; Nakajima et al. 2009; Gackowska et al. 2014)

Fig. 5-8 Exemplary Chromatograms of transformation products

Fig. 5 Gas chromatograms of the products of EHMC conversion in: a) EHMC/H_2_O_2_/UV [1:10] system and in b) EHMC/H2O2/HCl/UV system [1:10:10] (Gackowska et al. 2014)

Fig. 6 Exemplary gas chromatograms of EHMC/NaOCl (Gackowska et al. 2016)

Fig. 7 Exemplary gas chromatograms of EHMC/ NaOCl /UV (Gackowska et al. 2016)


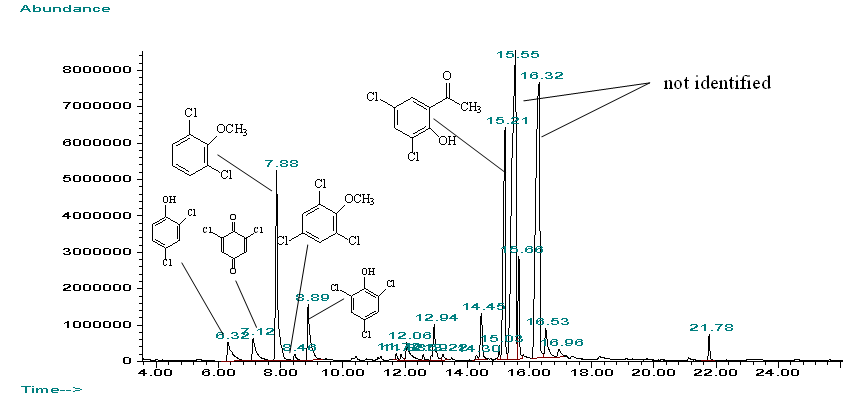
Fig. 8 Exemplary gas chromatograms of MCA/NaOCl/UV (Gackowska et al. 2016)


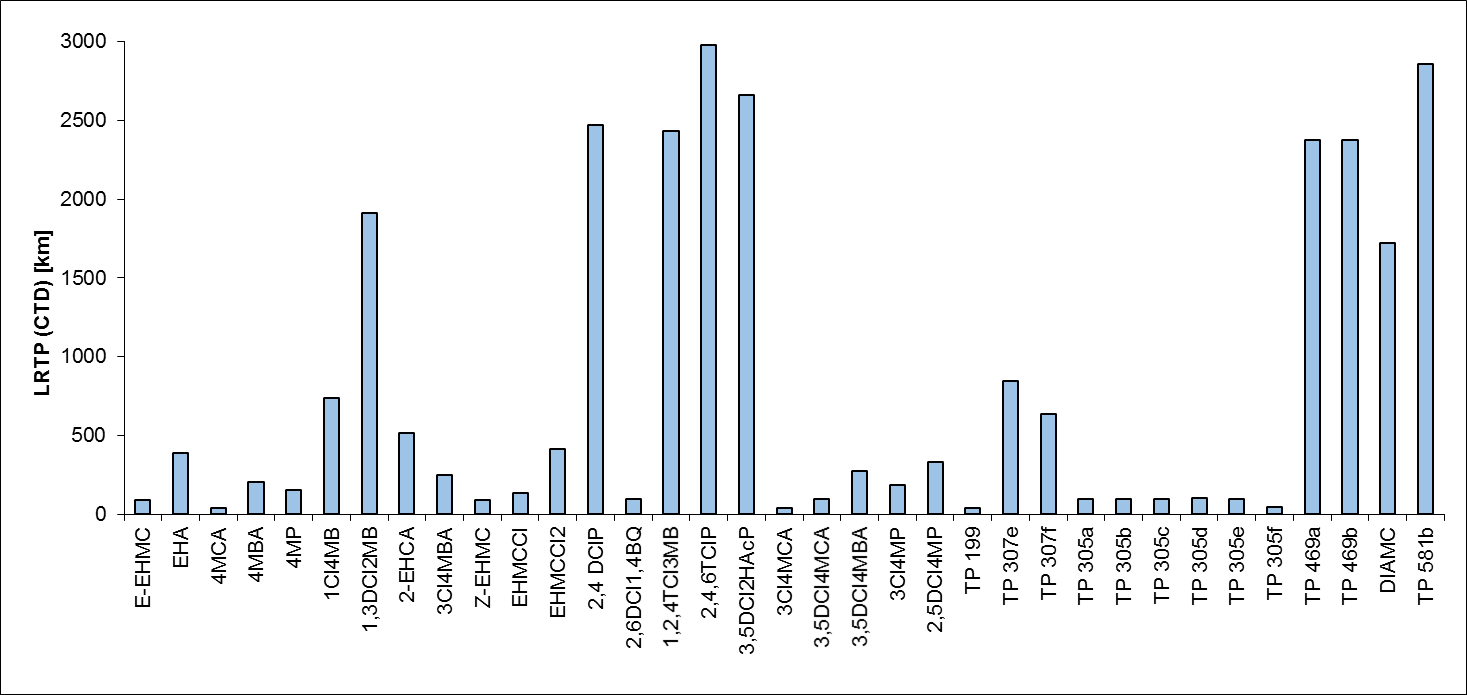


Fig. 9 Estimated values of long-range transport potential (LRTP) (expressed by Characteristic Travel Distance (CTD)) of all EHMC transformation products


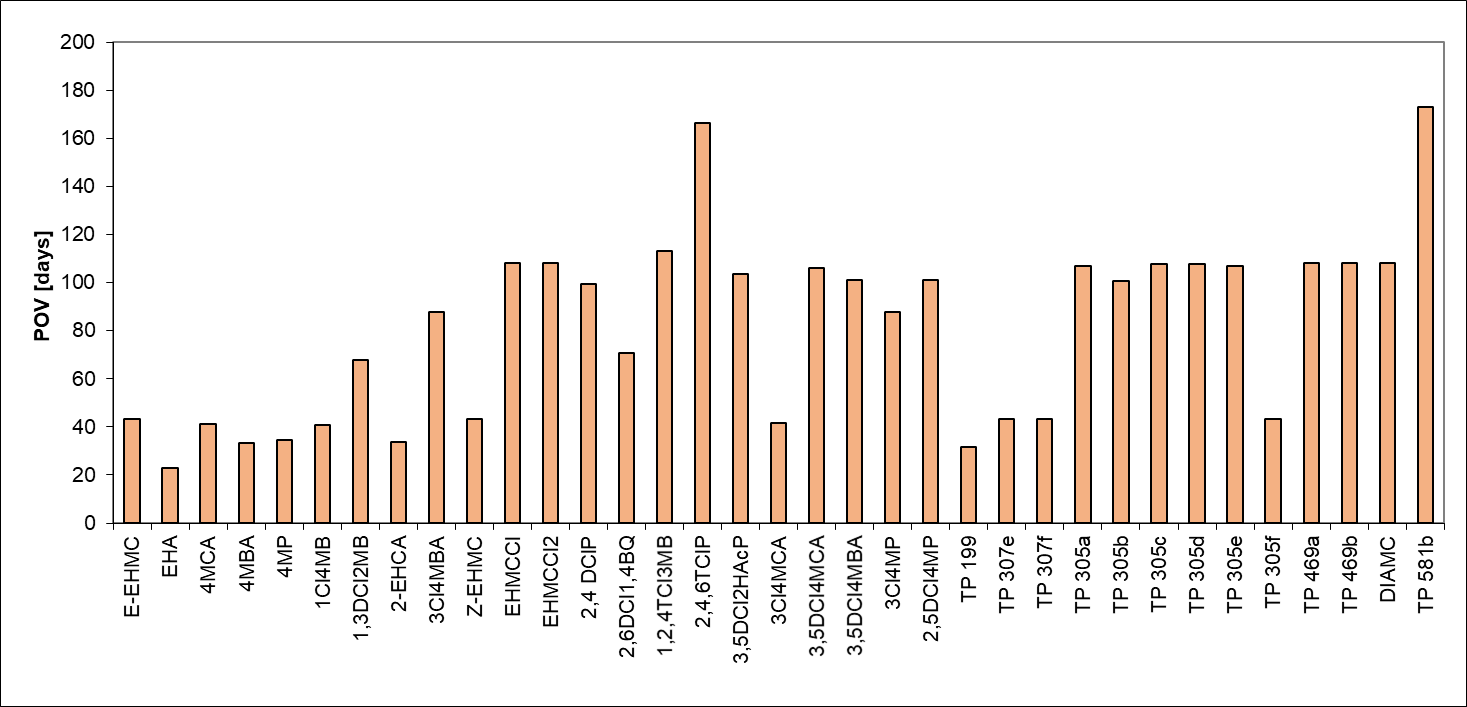


Fig. 10 Estimated values of the overall persistence (P_ov_) of all EHMC transformation products


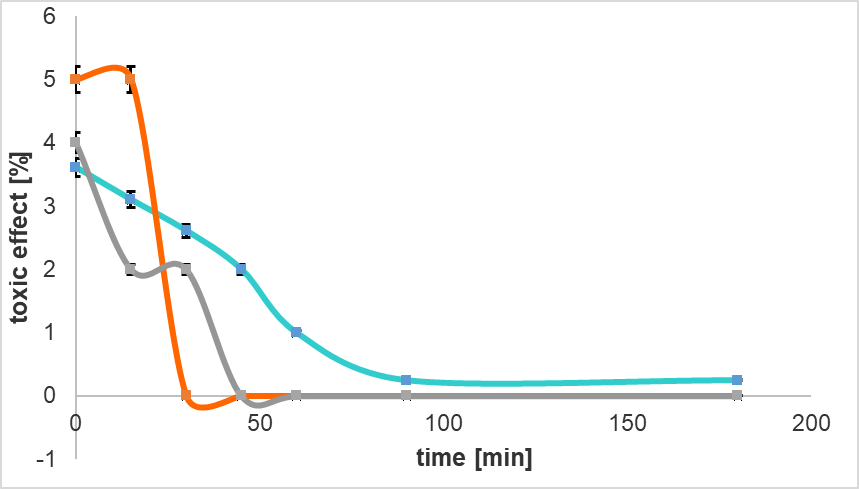


Fig. 11 Toxic effect of the studied system NaOCl/UV determined over time using different toxicity tests: ―▪―*Microtox*; ―▪― *Daphtoxkit* ; ―▪― *Artoxkit*


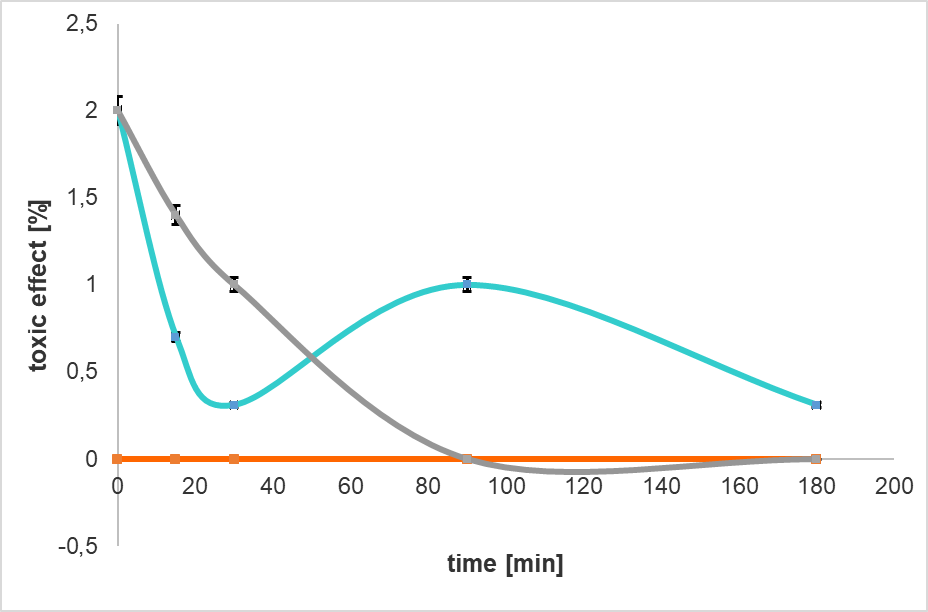


Fig. 12 Toxic effect of the studied system H_2_O_2_/UV determined over time using different toxicity tests : ―▪―*Microtox*; ―▪― *Daphtoxkit* ; ―▪― *Artoxkit*


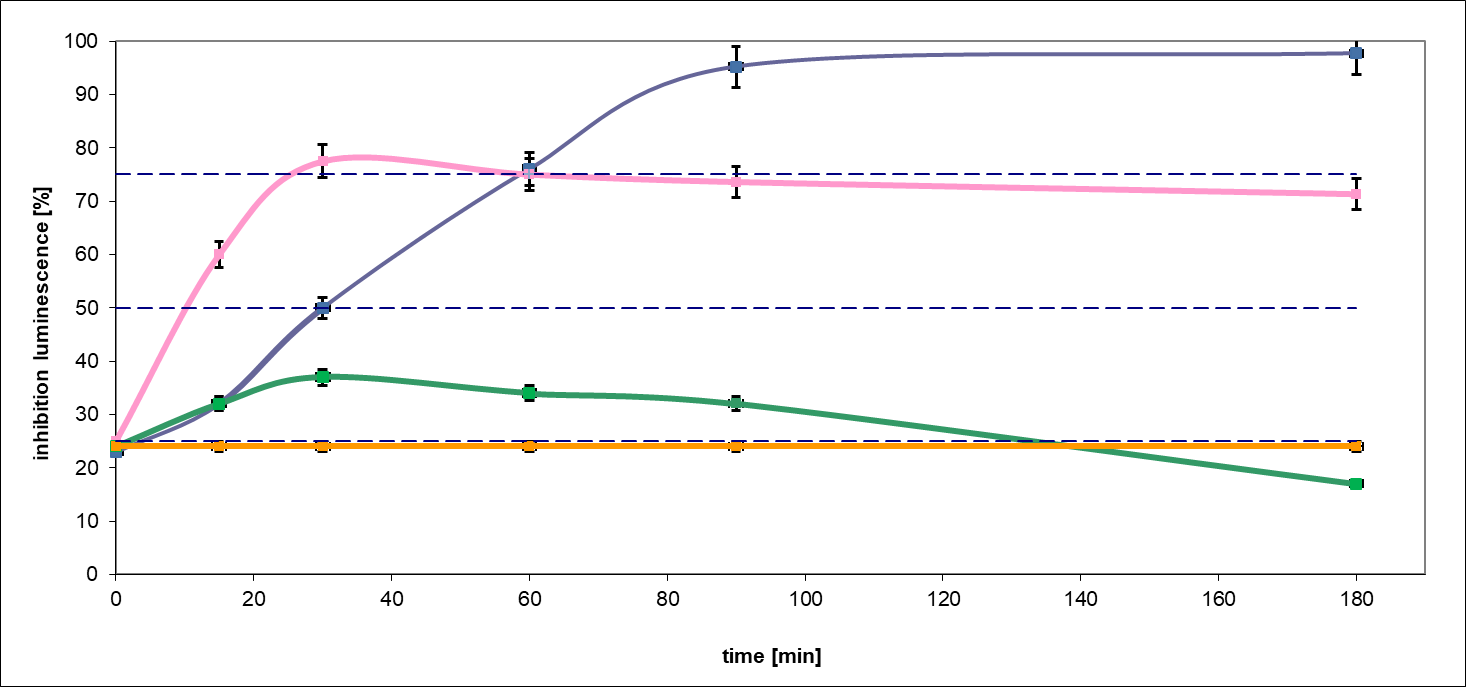


Fig. 13 Toxic effect of the studied systems determined over time using Microtox® test (after 5 minutes of exposure): ―▪―EHMC/NaOCl/UV; ―▪― EHMC/H_2_O_2_/UV; ―▪― EHMC/UV; ―▪― EHMC


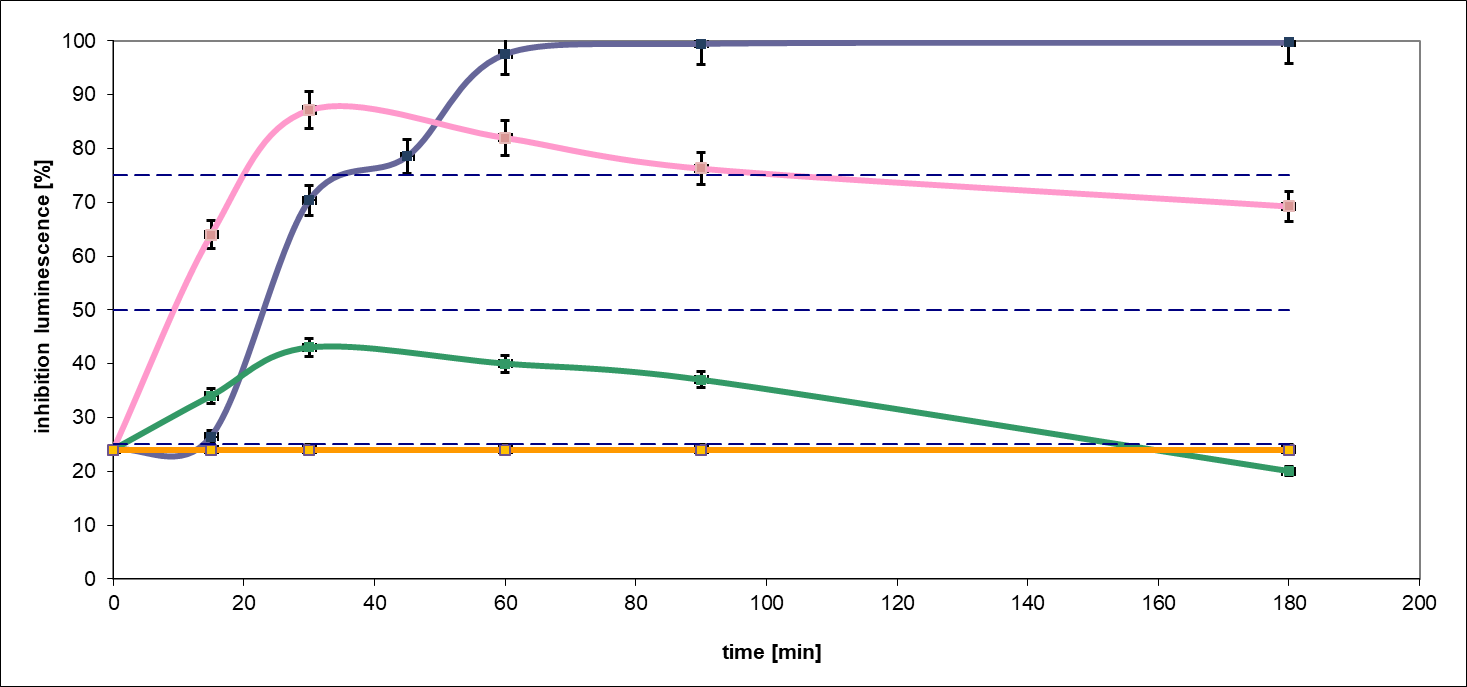


Fig. 14 Toxic effect of the studied systems determined over time using Microtox® test (after 15 minutes of exposure): ―▪―EHMC/NaOCl/UV; ―▪― EHMC/H_2_O_2_/UV; ―▪― EHMC/UV; ―▪― EHMC


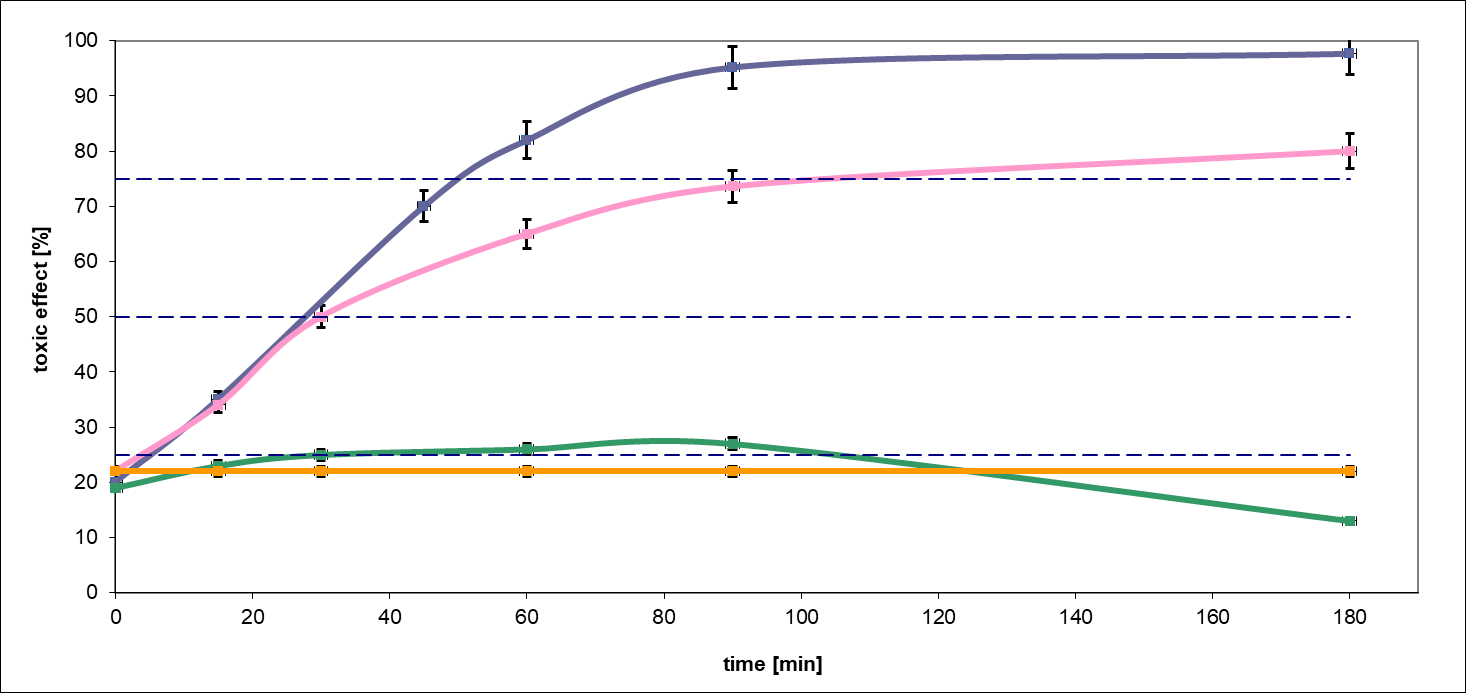


Fig. 15 Toxic effect of the studied systems determined over time using *Artoxkit M®* test (after 24 h of exposure): ―▪―EHMC/NaOCl/UV; ―▪― EHMC/H_2_O_2_/UV; ―▪― EHMC/UV; ―▪― EHMC


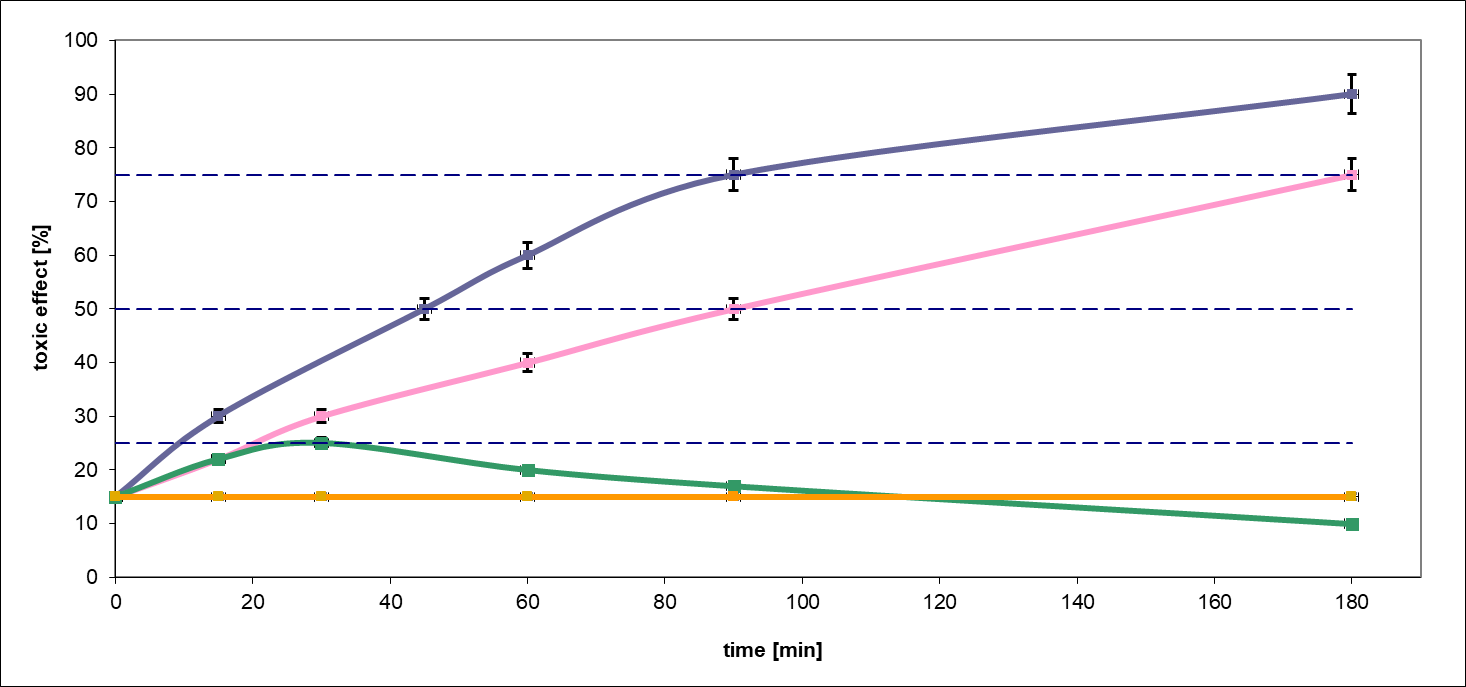


Fig. 16 Toxic effect of the studied systems determined over time using *Daphtoxkit F*® test (after 24 h of exposure): ―▪―EHMC/NaOCl/UV; ―▪― EHMC/H_2_O_2_/UV; ―▪― EHMC/UV; ―▪― EHMC


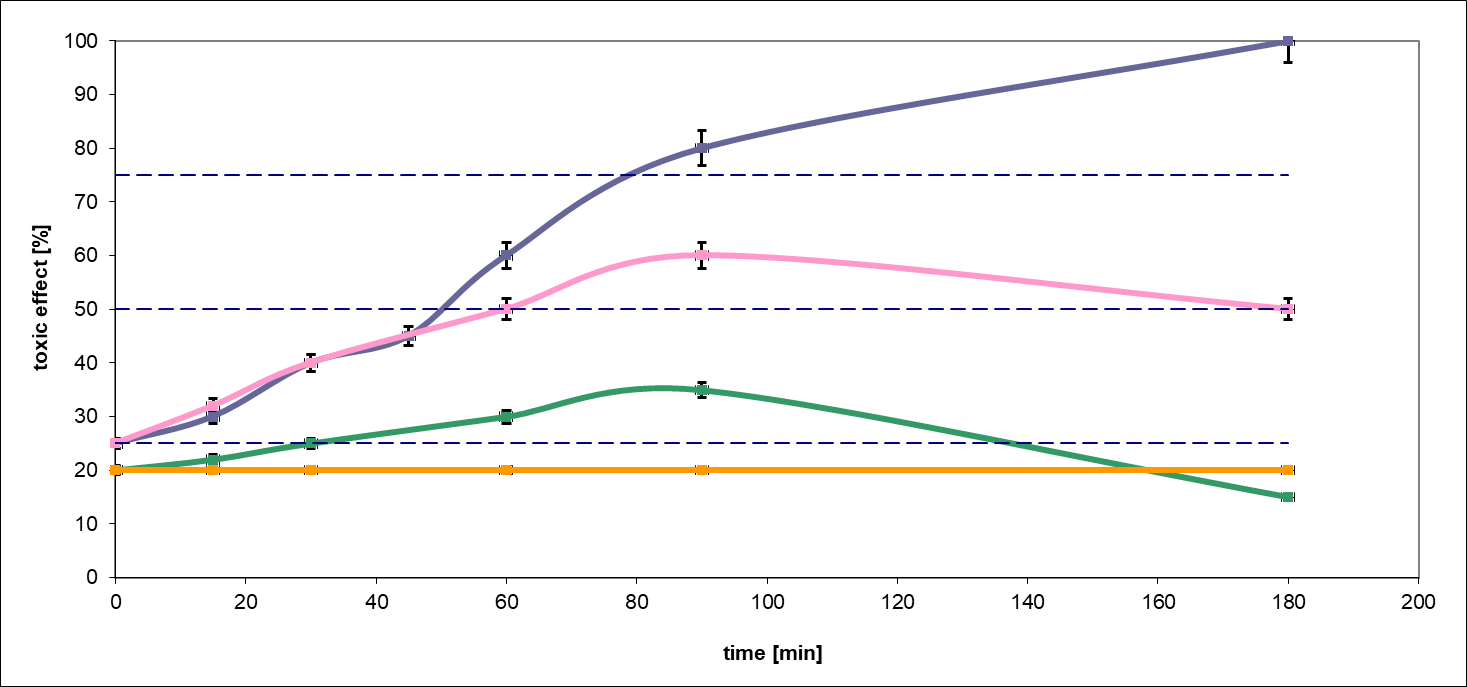


Fig. 17 Toxic effect of the studied systems determined over time using *Daphtoxkit F®* test (after 28 h of exposure): ―▪―EHMC/NaOCl/UV; ―▪― EHMC/H_2_O_2_/UV; ―▪― EHMC/UV; ―▪― EHMC
